# High pre-pregnancy BMI with a history of gestational diabetes mellitus is associated with an increased risk of type 2 diabetes in Korean women

**DOI:** 10.1371/journal.pone.0252442

**Published:** 2021-06-04

**Authors:** Dayeon Shin, Kyung Won Lee

**Affiliations:** 1 Department of Food and Nutrition, Inha University, Incheon, Republic of Korea; 2 Department of Home Economics Education, Korea National University of Education, Cheongju, Republic of Korea; University of Mississippi Medical Center, UNITED STATES

## Abstract

Despite the importance of pre-pregnancy body mass index (BMI) and a history of gestational diabetes mellitus (GDM) in the progression of GDM to type 2 diabetes, few studies have evaluated the combined effect of high pre-pregnancy BMI and GDM status on the future development of type 2 diabetes in Korean women. This study aimed to examine the relationship of pre-pregnancy BMI and GDM history with the risk of type 2 diabetes among Korean women. In addition, the effects of pre-pregnancy BMI and current BMI on the risk of type 2 diabetes were evaluated. Women who gave birth in the Health Examinees Study of the Korean Genome and Epidemiology Study from 2004 to 2013 (n = 59,258) were included in this study. Multivariable logistic regression was used to examine the association of pre-pregnancy BMI categories (underweight: <18.5 kg/m^2^; normal: 18.5–22.9 kg/m^2^; overweight: 23.0–24.9 kg/m^2^; obese: ≥25.0 kg/m^2^) and GDM history with the risk of type 2 diabetes after controlling for the following covariates: age, education, income, smoking status before the first pregnancy, alcohol consumption, regular exercise, menarche age, first pregnancy age, and first pregnancy outcome. Compared to women with normal pre-pregnancy BMIs, women with overweight and obese pre-pregnancy BMIs had higher odds of developing type 2 diabetes (adjusted odds ratio [AOR]: 1.13, 95% confidence interval [CI]: 1.02–1.25 and AOR: 1.29, 95% CI: 1.10–1.50, respectively) after controlling for covariates. Women with pre-pregnancy BMIs <23 kg/m^2^ and current BMIs ≥23 kg/m^2^ had increased odds of developing type 2 diabetes (AOR: 1.64, 95% CI: 1.51–1.78) compared to those with pre-pregnancy BMIs <23 kg/m^2^ and current BMIs <23 kg/m^2^. Among women without a history of GDM, those with overweight and obese pre-pregnancy BMIs had increased odds of developing type 2 diabetes compared to those with normal pre-pregnancy BMIs (AOR: 1.12, 95% CI: 1.01–1.24 and AOR: 1.23, 95% CI: 1.05–1.44, respectively). Among women with GDM, those with obese pre-pregnancy BMIs had increased odds of developing type 2 diabetes (AOR: 3.84, 95% CI: 1.52–9.87). This study showed that there was a higher likelihood of developing type 2 diabetes in women who were overweight or obese before pregnancy with a history of GDM compared to their counterparts without a history of GDM. Furthermore, high pre-pregnancy BMI or high current BMI increased the risk of type 2 diabetes in Korean women, regardless of GDM history. This emphasizes the importance of maintaining a healthy weight status before and after pregnancy to prevent the future risk of type 2 diabetes.

## Introduction

Gestational diabetes mellitus (GDM) is defined as glucose intolerance that is first identified during pregnancy [1], and its prevalence has increased globally over the past 10–20 years [2, 3]. GDM, one of the most common pregnancy complications, affected 5.7% to 9.5% in Korea in 2009 and 2011, according to data from the Health and Insurance Review and Assessment database [4]. The Korean national guidelines during that period recommended all pregnant women to perform GDM screening at 24–28 weeks, regardless of underlying GDM risk [5]. GDM influences short- and long-term adverse health outcomes for both mothers and their offspring [6, 7]. A huge economic burden and significant increase in health care costs associated GDM have been documented [8, 9].

Using more than 4 years of follow-up longitudinal study, women with a history of GDM were found to have a faster deterioration of insulin sensitivity over time, and their beta cell compensation continued to deteriorate at a faster rate during follow-up compared to those without a history of GDM [10]. Among women with GDM, the metabolic stress of pregnancy that they experience may unmask a genetic predisposition to type 2 diabetes [11], which may lead to chronic disease states such as impaired glucose levels and type 2 diabetes after pregnancy [12]. Specifically, it has been reported that women with a history of GDM have a 7-fold increased risk of type 2 diabetes. Thus, a diagnosis of GDM can be used to identify women and their offspring who may be more vulnerable to diabetes, obesity, and cardiovascular disease later in their lives [12–16]. The most important modifiable risk factor for GDM is pre-pregnancy body mass index (BMI) [17, 18]. A previous study showed that every unit increase in pre-pregnancy BMI was associated with significantly shorter cord blood telomere length and placental telomere length in a large birth cohort [19]. This indicated that pre-pregnancy weight might influence longevity in the offspring. Furthermore, recent findings suggest that pre-pregnancy BMI is associated with the development of regulation of body weight and thalamic functional brain connectivity in the offspring [20]. Pre-pregnancy BMI may be one of the most important modifiable environmental factors that may dictate the life expectancy of and brain development in newborns, and their later health status and risk of chronic diseases [19, 20].

Despite the importance of the influence of pre-pregnancy BMI, current BMI, and GDM history on the development of type 2 diabetes, limited studies have been conducted to evaluate the combined effect of high pre-pregnancy BMI and GDM status on the development of type 2 diabetes in a large sample of Korean women. Therefore, we examined the relationship of pre-pregnancy BMI and GDM history with the risk of type 2 diabetes. We further explored the combined effects of pre-pregnancy BMI and current BMI on the future development of type 2 diabetes.

## Material and methods

### Study participants

Study participants were recruited from the Health Examinees Study (HEXA) of the Korean Genome and Epidemiology Study (KoGES), a large-scale, community-based prospective cohort study. We used baseline examinations, which were obtained from 2004 to 2013 by the Korea Centers for Disease Control and Prevention. A total of 173,208 study participants aged ≥40 years were recruited from 38 major hospitals and local health examination centers in eight regions of Korea. The HEXA study design has been described in detail elsewhere [21, 22]. An interview-based questionnaire survey was conducted among women to collect information on sociodemographic characteristics, medical history, medication usage, family history, lifestyle factors, diet, physical activity, and reproductive factors. The KoGES study was reviewed and approved by the Institutional Review Board of the Korea Centers for Disease Control and Prevention. All participants enrolled in the study voluntarily and all gave written-informed consent. All study methods and protocols were conducted in accordance with the relevant institutional guidelines and regulations. The study protocol was reviewed and approved by the Institutional Review Board (IRB) of Inha University on January 31, 2020 (IRB No. 200129–1A).

**Fig 1:**
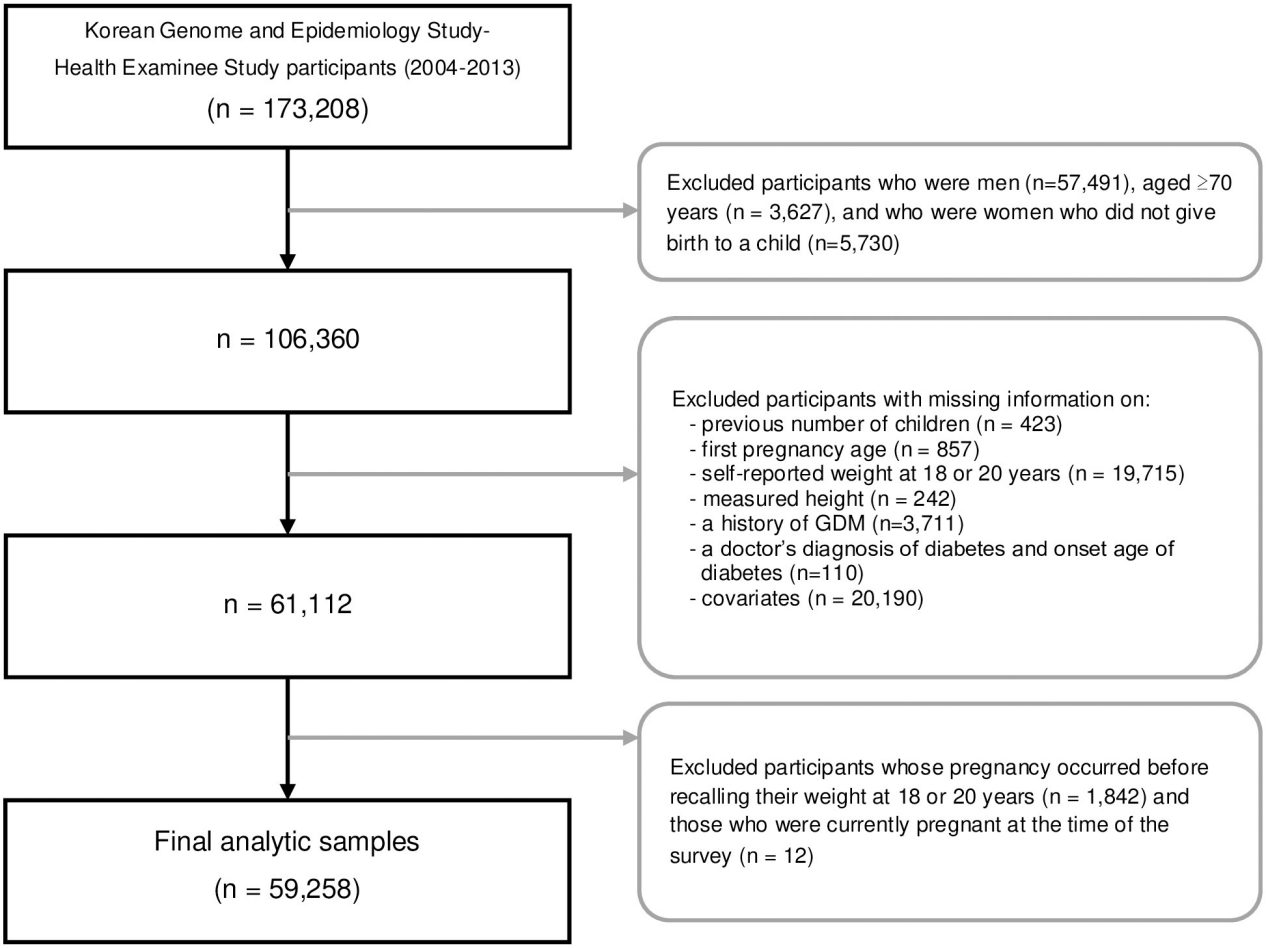
Flow chart of study population.

Fig 1 presents flow chart of study participants available in KoGES. At baseline, 173,208 individuals participated in the HEXA study. We excluded participants who were men, aged ≥70 years, and who were women who did not give birth to a child. We also excluded participants with missing information on previous number of children, first pregnancy age, self-reported weight at 18 or 20 years, measured height, a history of GDM, and a doctor’s diagnosis of diabetes and onset age of diabetes, and covariates. Further, we excluded participants whose pregnancy occurred before recalling their weight at 18 or 20 years and those who were currently pregnant at the time of the survey. The final study sample comprised 59,258 women.

### Pre-pregnancy BMI

In the surveys from 2004 to 2006, women had to report their weight at age 20, and in the surveys from 2007 to 2012, women were asked to report their weight at age 18. BMI was calculated by dividing the recalled weight at age 18 or 20, which was before their first childbirth, by the measured height at baseline squared. Pre-pregnancy BMI was categorized as “underweight (<18.5 kg/m^2^),” “normal weight (18.5–22.9 kg/m^2^),” “overweight (23.0–24.9 kg/m^2^)”, and “obese (≥25.0 kg/m^2^)”, according to the criteria for Asians [23].

### Current BMI

Current BMI was calculated by dividing the measured weight by the measured height at baseline squared (kg/m^2^) at the time of the survey.

### Ascertainment of GDM and type 2 diabetes

A woman was considered to have GDM if they responded “yes” to the following question: “have you ever been diagnosed with GDM in the past?” Type 2 diabetes was defined as a woman diagnosed with diabetes by a doctor or a fasting blood glucose level ≥126 mg/dL. After fasting overnight for 12 hours, plasma concentrations of glucose were measured enzymatically using a HITACHI Automatic Analyzer 7600 (Hitachi, Tokyo, Japan).

### Confounding factors

Confounding factors included sociodemographic, lifestyle, and reproductive variables. Sociodemographic variables included age, education level, and monthly income. Lifestyle variables included smoking before the first pregnancy, current alcohol consumption, and regular exercise. Reproductive variables included age at menarche, age at first pregnancy, first pregnancy outcome, and number of children. Education level was grouped into three categories: “≤high school,” “vocational school graduate or college dropout,” and “college and above”. Monthly income was classified into four categories: “<1 million Korean won,” “1–2 million Korean won”, “2–3 million Korean won”, and “3 million Korean won and above.” Smoking status before the first pregnancy was classified as “yes” or “no.” Alcohol consumption status was classified into three categories: “non-drinker,” “past drinker,” and “current drinker.” Regular exercise was categorized as “yes (active)” or “no (inactive)”.

### Statistical analyses

Descriptive statistics were computed to detect significant differences between the variables according to the status of type 2 diabetes using chi-square tests for categorical variables and t-tests for continuous variables. Multivariable logistic regression was used to compute odds ratios and 95% confidence intervals of the associations of pre-pregnancy BMI with GDM and type 2 diabetes after controlling for confounders. Confounding variables included age, education, income, smoking status before the first pregnancy, alcohol consumption, regular exercise, menarche age, first pregnancy age, and first pregnancy outcome. Furthermore, we stratified the associations between pre-pregnancy BMI and the risk of type 2 diabetes according to the status of GDM history. All the statistical analyses were conducted using SAS version 9.4 (SAS Institute, Cary, NC, USA). A two-sided *P* value of <0.05 was considered statistically significant.

## Results

Table 1 shows the sociodemographic, lifestyle, and reproductive variables according to the status of type 2 diabetes. Age, education, monthly income, alcohol consumption, regular exercise, menarche age, first pregnancy age, first pregnancy outcome, number of children, and pre-pregnancy BMI significantly differed according to the status of type 2 diabetes. Women with type 2 diabetes were significantly older (57.0 ± 7.1 vs. 51.9 ± 7.5 years), had lower education levels (≤high school: 87.9% vs. 75.1%), had lower monthly income levels (<1 million Korean won: 20.2% vs. 9.75%), were more likely to be non-drinkers of alcohol (77.0% vs. 65.9%), were more likely to engage in regular exercise (54.3% vs. 51.5%), had higher menarche ages (≥16 years: 47.8% vs. 37.7%), had lower first pregnancy ages (≤23 years: 41.7% vs. 30.4%), had more live births (83.9% vs. 81.7%), had more children (2.7 ± 0.9 vs. 2.4 ± 0.7), and higher pre-pregnancy BMIs (21.0 ± 2.4 vs. 20.6 ± 2.2 kg/m^2^) than those without type 2 diabetes.

**Table 1:** Distributions of sociodemographic, lifestyle, and reproductive characteristics of the study population in the Health Examinee Study (HEXA) 2004–2013.

	Type 2 Diabetes				Total		*P* value
	No		Yes				
	Mean	SD	Mean	SD	Mean	SD	
Age (years)	51.9	7.5	57.0	7.1	52.2	7.6	< .0001
Number of children	2.4	0.7	2.7	0.9	2.4	0.7	< .0001
Pre-pregnancy BMI (kg/m^2^)	20.6	2.2	21.0	2.4	20.6	2.2	< .0001
	**n**	**%**	**n**	**%**	**n**	**%**	
Education							
≤High school	41,718	75.1	3,279	87.9	44,997	75.9	< .0001
Vocational school graduate or college dropout	2,593	4.7	110	3.0	2,703	4.6	
≥College	11,215	20.2	343	9.2	11,558	19.5	
Monthly income							
<1 million Korean won	5,414	9.8	754	20.2	6,168	10.4	< .0001
1–2 million Korean won	11,048	19.9	1,018	27.3	12,066	20.4	
2–3 million Korean won	12,631	22.8	805	21.6	13,436	22.7	
≥3 million Korean won	26,433	47.6	1,155	31.0	27,588	46.6	
Smoking before the first pregnancy							
No	55,157	99.3	3,716	99.6	58,873	99.4	0.0826
Yes	369	0.7	16	0.4	385	0.7	
Alcohol consumption							
Never	36,589	65.9	2,875	77.0	39,464	66.6	< .0001
Past	913	1.6	79	2.1	992	1.7	
Current	18,024	32.5	778	20.9	18,802	31.7	
Regular exercise							
No	26,933	48.5	1,706	45.7	28,639	48.3	0.001
Yes	28,593	51.5	2,026	54.3	30,619	51.7	
Menarche age (years)							
≤14	21,789	39.2	1,165	31.2	22,954	38.7	< .0001
15	12,794	23.0	784	21.0	13,578	22.9	
≥16	20,943	37.7	1,783	47.8	22,726	38.4	
First pregnancy age (years)							
≤23	16,853	30.4	1,555	41.7	18,408	31.1	< .0001
24–26	23,227	41.8	1,427	38.2	24,654	41.6	
≥27	15,446	27.8	750	20.1	16,196	27.3	
First pregnancy outcome							
Live birth	45,348	81.7	3,132	83.9	48,480	81.8	0.0001
Stillbirth	364	0.7	29	0.8	393	0.7	
Extrauterine pregnancy	99	0.2	7	0.2	106	0.2	
Spontaneous abortion	3,900	7.0	264	7.1	4,164	7.0	
Artificial abortion	5,815	10.5	300	8.0	6,115	10.3	

*P* values based on t-tests for continuous variables and chi-square tests for categorical variables.

Table 2 shows the associations of pre-pregnancy BMI with the risk of GDM and type 2 diabetes. Compared to women with normal pre-pregnancy BMIs, those with obese pre-pregnancy BMIs had higher odds of GDM (AOR: 1.63, 95% CI: 1.05–2.55) after controlling for the following covariates: age, education, income, smoking status before the first pregnancy, alcohol consumption, physical activity, menarche age, first pregnancy age, and first pregnancy outcome. Compared to women with normal pre-pregnancy BMIs, those with overweight and obese pre-pregnancy BMIs had higher odds of developing type 2 diabetes (AOR: 1.13, 95%: 1.02–1.25 and AOR: 1.29, 95% CI: 1.10–1.50, respectively) after controlling for the same covariates.

**Table 2:** Associations of pre-pregnancy BMI with GDM and type 2 diabetes in the Health Examinee Study (HEXA) 2004–2013.

Pre-pregnancy BMI (kg/m^2^)	GDM	Type 2 Diabetes
Underweight (<18.5)	0.95 (0.77–1.17)	0.99 (0.89–1.09)
Normal (18.5–22.9)	1.00 (Ref.)	1.00 (Ref.)
Overweight (23–24.9)	1.19 (0.91–1.56)	1.13 (1.02–1.25)
Obese (≥25)	1.63 (1.05–2.55)	1.29 (1.10–1.50)

Data are presented as adjusted odds ratio (95% confidence intervals) after controlling for age (continuous), education (≤high school, vocational school, graduate or college dropout, ≥college), income (<1 million won, 1–2 million Korean won, 2–3 million Korean won, ≥3 million Korean won), smoking status before the first pregnancy (no, yes), alcohol consumption (never, past, current), regular exercise (no, yes), menarche age (≤14, 15, and ≥16 years), first pregnancy age (≤23, 24–26, and ≥27 years), and first pregnancy outcome (live birth, stillbirth, extrauterine pregnancy, spontaneous abortion, artificial abortion).

GDM: Gestational diabetes mellitus; BMI: Body mass index.

Table 3 presents the distributions of pre-pregnancy BMI categories and risk of type 2 diabetes according to the status of GDM history. The associations between pre-pregnancy BMI and type 2 diabetes according to the GDM status are also presented. Among women with and without GDM, most women had normal pre-pregnancy BMIs (69.2% and 70.8%, respectively). Among women without GDM, those with overweight and obese pre-pregnancy BMIs had higher odds of developing type 2 diabetes than those with normal pre-pregnancy BMIs (AOR: 1.12, 95% CI: 1.01–1.24 and AOR: 1.23, 95% CI: 1.05–1.44, respectively). Among women with GDM, those with obese pre-pregnancy BMIs had higher odds of developing type 2 diabetes than those with normal pre-pregnancy BMIs (AOR: 3.87, 95% CI: 1.52–9.87).

**Table 3:** Association between pre-pregnancy BMI and type 2 diabetes according to the status of GDM history in the Health Examinee Study (HEXA) 2004–2013.

Pre-pregnancy BMI (kg/m^2^)	Without a history of GDM (n = 58,629)			With a history of GDM (n = 629)		
	n	%	AOR (95% CI)	n	%	AOR (95% CI)
Underweight (<18.5)	9,402	16.0	0.99 (0.89–1.09)	111	17.7	1.13 (0.65–1.97)
Normal (18.5–22.9)	41,429	70.8	1.00 (Ref.)	435	69.2	1.00 (Ref.)
Overweight (23.0–24.9)	6,002	10.2	1.12 (1.01–1.24)	62	9.9	1.43 (0.75–2.73)
Obese (≥25.0)	1,796	3.1	1.23 (1.05–1.44)	21	3.3	3.87 (1.52–9.87)

Data are presented as numbers, percentages, and adjusted odds ratio (95% confidence intervals) after controlling for age (continuous), education (≤high school, vocational school, graduate or college dropout, ≥college), income (<1 million Korean won, 1–2 million Korean won, 2–3 million Korean won, ≥3 million Korean won), smoking status before the first pregnancy (no, yes), alcohol consumption (never, past, current), regular exercise (no, yes), menarche age (≤14, 15, and ≥16 years), first pregnancy age (≤23, 24–26, and ≥27 years), and first pregnancy outcome (live birth, stillbirth, extrauterine pregnancy, spontaneous abortion, artificial abortion).

GDM: Gestational diabetes mellitus; BMI: Body mass index; AOR: Adjusted odds ratio; CI: Confidence interval.

**Fig 2:**
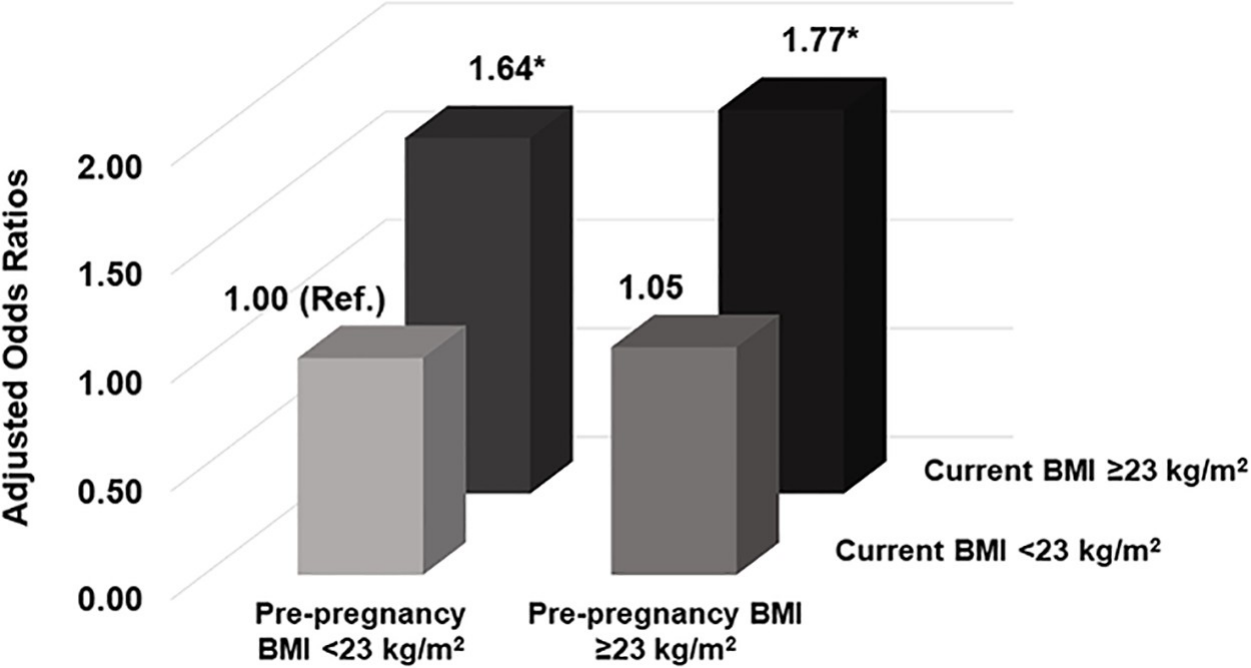
Associations of pre-pregnancy BMI and current BMI with the development of type 2 diabetes in the in the Health Examinee Study (HEXA) 2004–2013. Data are presented as adjusted odds ratios after controlling for age (continuous), education (≤high school, vocational school, graduate or college dropout, ≥college), income (<1 million Korean won, 1–2 million Korean won, 2–3 million won, ≥3 million Korean won), smoking status before the first pregnancy (no, yes), alcohol consumption (never, past, current), regular exercise (no, yes), menarche age (≤14, 15, and ≥16 years), first pregnancy age (≤23, 24–26, and ≥27 years), and first pregnancy outcome (live birth, stillbirth, extrauterine pregnancy, spontaneous abortion, artificial abortion). *P value <0.05. BMI: Body mass index.

Table 4 shows the combined effects of pre-pregnancy BMI and current BMI on type 2 diabetes. The distributions of the combined effects of pre-pregnancy BMI and current BMI significantly differed according to the status of type 2 diabetes (*P* <0.0001). A high proportion of women with type 2 diabetes had pre-pregnancy BMI ≥23 kg/m^2^ and current BMI ≥23 kg/m^2^ (10.0%). The distribution of type 2 diabetes significantly differed according to the pre-pregnancy BMI and current BMI categories (*P* <0.0001). The association of the combined effects of pre-pregnancy BMI and current BMI with the risk of type 2 diabetes is presented (Fig 2). Women with pre-pregnancy BMIs <23 kg/m^2^ and current BMIs ≥23 kg/m^2^ had higher odds of developing type 2 diabetes (AOR: 1.64, 95% CI: 1.51–1.78) than those with pre-pregnancy BMIs <23 kg/m^2^ and current BMI <23 kg/m^2^. In addition, women with pre-pregnancy BMIs ≥23 kg/m^2^ and current BMIs ≥23 kg/m^2^ had higher odds of developing type 2 diabetes (AOR 1.77, 95% CI 1.59–1.98) than those with pre-pregnancy BMIs <23 kg/m^2^ and current BMIs <23 kg/m^2^.

**Table 4:** Distributions of pre-pregnancy BMI and current BMI categories according to the status of type 2 diabetes in the Health Examinee Study (HEXA) 2004–2013.

BMI categories	Type 2 diabetes						*P* value
	No		Yes		Total		
	n	%	n	%	n	%	
Pre-pregnancy BMI <23 kg/m^2^ & Current BMI <23 kg/m^2^	23,921	96.1	969	3.9	24,890	42.0	<0.0001
Pre-pregnancy BMI ≥23 kg/m^2^ & Current BMI <23 kg/m^2^	1,709	94.6	98	5.4	1,807	3.1	
Pre-pregnancy BMI <23 kg/m^2^ & Current BMI ≥23 kg/m^2^	24,423	92.2	2,059	7.8	26,482	44.7	
Pre-pregnancy BMI ≥23 kg/m^2^ & Current BMI ≥23 kg/m^2^	5,467	90.0	606	10.0	6,073	10.3	
Total	55,520	93.7	3,732	6.3	59,252	100.0	

*P* value based on the chi-square test.

BMI: Body mass index.

Table 5 demonstrates the associations of current BMI and combined effects of pre-pregnancy BMI and current BMI with type 2 diabetes according to the status of GDM history. A higher proportion of women with a history of GDM had obese current BMIs than those without a history of GDM (30.2% vs. 28.0%). Among women without a history of GDM, those with overweight or obese current BMIs had a higher risk of type 2 diabetes than those with normal current BMIs (AOR: 1.29, 95% CI: 1.17–1.41 and AOR: 1.96, 95% CI: 1.81–2.14, respectively). Among women with a history of GDM, those with obese current BMIs were 2.04 more likely to develop type 2 diabetes (AOR: 2.04, 95% CI: 1.24–3.35) than those with normal current BMIs. Among women without a history of GDM, those with pre-pregnancy BMIs <23 kg/m^2^ and current BMIs ≥23 kg/m^2^ as well as those with pre-pregnancy BMIs ≥23 kg/m^2^ and current BMIs ≥23 kg/m^2^ had a higher risk of type 2 diabetes than those with pre-pregnancy BMIs ≥23 kg/m^2^ and current BMIs <23 kg/m^2^ (AOR: 1.63, 95% CI: 1.50–1.77; AOR: 1.73, 95% CI: 1.55–1.93, respectively). Among women with a history of GDM, those with pre-pregnancy BMIs ≥23 kg/m^2^ and current BMIs ≥23 kg/m^2^ had a higher risk of type 2 diabetes than those with pre-pregnancy BMIs ≥23 kg/m^2^ and current BMIs <23 kg/m^2^ (AOR: 2.54, 95% CI: 1.32–4.89).

**Table 5:** Association between current BMI and type 2 diabetes according to the status of GDM history in the Health Examinee Study (HEXA) 2004–2013.

	**Without a history of GDM** (**n** = **58,623**)			**With a history of GDM** (**n** = **629**)		
**Current BMI** (**kg/m**^**2**^)	**n**	**%**	**AOR (95% CI)**	**n**	**%**	**AOR (95% CI)**
Underweight (<18.5)	1,072	1.8	0.86 (0.60–1.23)	15	2.4	0.53 (0.06–4.34)
Normal (18.5–22.9)	25,365	43.3	1.00 (Ref.)	245	39.0	1.00 (Ref.)
Overweight (23.0–24.9)	15,768	26.9	1.29 (1.17–1.41)	179	28.5	1.06 (0.63–1.80)
Obese (≥25.0)	16,418	28.0	1.96 (1.81–2.14)	190	30.2	2.04 (1.24–3.35)
**BMI categories**	**n**	**%**	**AOR (95% CI)**	**n**	**%**	**AOR (95% CI)**
Pre-pregnancy BMI <23 kg/m^2^ & Current BMI <23 kg/m^2^	24,646	42.0	1.00 (Ref.)	244	38.8	1.00 (Ref.)
Pre-pregnancy BMI ≥23 kg/m^2^ & Current BMI <23 kg/m^2^	1,791	3.1	1.03 (0.82–1.28)	16	2.5	1.46 (0.42–5.04)
Pre-pregnancy BMI <23 kg/m^2^ & Current BMI ≥23 kg/m^2^	26,180	44.7	1.63 (1.50–1.77)	302	48.0	1.41 (0.89–2.25)
Pre-pregnancy BMI ≥23 kg/m^2^ & Current BMI ≥23 kg/m^2^	6,066	10.3	1.73 (1.55–1.93)	67	10.7	2.54 (1.32–4.89)

Data are presented as numbers, percentages, and adjusted odds ratios (95% confidence intervals) after controlling for age (continuous), education (≤high school, vocational school, graduate or college dropout, ≥college), income (<1 million won, 1–2 million Korean won, 2–3 million Korean won, ≥3 million Korean won), smoking status before the first pregnancy (no, yes), alcohol consumption (never, past, current), regular exercise (no, yes), menarche age (≤14, 15, and ≥16 years), first pregnancy age (≤23, 24–26, and ≥27 years), and first pregnancy outcome (live birth, stillbirth, extrauterine pregnancy, spontaneous abortion, artificial abortion).

GDM: Gestational diabetes mellitus; BMI: Body mass index; AOR: Adjusted odds ratio; CI: Confidence interval.

## Discussion

This study aimed to examine the associations of pre-pregnancy BMI, current BMI, and GDM history with type 2 diabetes in Korean women. We examined the relationships of pre-pregnancy BMI and GDM with type 2 diabetes. Among 59,258 women, pre-pregnancy BMI was associated with an increased risk of GDM and type 2 diabetes. In accordance with our findings, using data from the U.S. Pregnancy Risk Assessment Monitoring System, women with obese pre-pregnancy BMIs (≥30 kg/m^2^) had increased odds of GDM (AOR: 2.78, 95% CI: 2.60–2.96) [18]. In a retrospective longitudinal cohort study from the U.S., the prevalence of GDM was higher in women who were obese (17.1%) or overweight (12.0%) before pregnancy than in those who had normal weights (7.4%) [24]. A meta-analysis [25] that included 70 studies involving 671,945 women revealed that those who were overweight, moderately obese, and morbidly obese before pregnancy had higher risks of GDM (OR: 1.97, 95% CI: 1.77–2.19; OR: 3.01, 95% CI: 2.34–3.87; and OR: 5.55, 95% CI: 4.27–7.21, respectively).

When stratified by GDM history, women with pre-pregnancy obesity and a history of GDM were 3.87 times more likely to develop type 2 diabetes than those with normal pre-pregnancy BMIs. However, women with pre-pregnancy obesity and without a history of GDM were 1.23 times more likely to develop type 2 diabetes, which was less than one-third of the odds of those with a history of GDM. This highlighted that the combination of pre-pregnancy obesity and GDM history may be associated with a greater likelihood of developing type 2 diabetes in the future.

Furthermore, we evaluated the combined effect of pre-pregnancy BMI and current BMI on the risk of type 2 diabetes. Interestingly, women with pre-pregnancy BMI <23 kg/m^2^ and current BMI ≥23 kg/m^2^ had 1.64-fold increased risk of type 2 diabetes compared to those women with both pre-pregnancy BMI and current BMI <23 kg/m^2^. When women were not overweight or obese before pregnancy, but became overweight or obese in their later lives, this increases the risk of type 2 diabetes emphasizing the role of current weight status. We found that the combination of pre-pregnancy BMI ≥23 kg/m^2^ and current BMI ≥23 kg/m^2^ was associated with a 1.77-fold increased risk of type 2 diabetes compared to pre-pregnancy BMI <23 kg/m^2^ and current BMI <23 kg/m^2^ after controlling for covariates. Both high BMI before pregnancy and high current BMI were associated with the risk of type 2 diabetes, and type 2 diabetes was closely linked with the presence of obesity before and after pregnancy. The association between obesity and type 2 diabetes has been well-established, and the major basis for this association is insulin resistance [26]. Insulin resistance in obesity and type 2 diabetes is caused by decreased insulin-stimulated glucose transport and metabolism in adipocytes [27]. Specifically, the impaired ability of insulin to lower blood glucose levels is partially due to disruptions in the translocation of the Glut4 glucose transporter to the surface membrane of muscle cells [28, 29] which in turn reduces glucose uptake from the bloodstream [30].

Weight status, especially obesity, plays a key role in the pathogenesis of type 2 diabetes [31, 32]. In line with our findings, previous studies have reported that past and present weight status are significantly associated with the risk of type 2 diabetes. In 2,927 Japanese adults with a mean age of 59.3 years, previous and current obesity were risk factors for diabetic nephropathy (OR: 1.66, 95% CI: 1.32–2.07 and OR: 2.48, 95% CI: 1.96–3.14, respectively) [33]. In a national cohort of 8,545 adults in the U.S. from the National Health and Nutrition Examination Survey Epidemiology Follow-up Study [34], the associations between weight change over approximately 10 years and the incidence of diabetes were investigated. Participants who gained 11–20 kg or more than 20 kg had a higher risk of developing diabetes (hazard ratio [HR]: 2.57, 95% CI: 1.84–3.85 and HR: 3.85, 95% CI: 2.04–7.22, respectively) than those whose weights remained stable over the 10-year period. Similarly, in Korean adults with a mean age of 56 years [35], a history of obesity and increased upper body adiposity were associated with non-insulin-dependent diabetes mellitus.

We further examined the associations of pre-pregnancy BMI and current BMI with type 2 diabetes stratified by GDM history. We found that women with a history of GDM, who were overweight or obese before pregnancy, and were currently obese were 2.54 times more likely to develop type 2 diabetes than those who were overweight or obese before pregnancy and currently had normal weights or were underweight. However, women with a history of GDM, who were overweight and obese before pregnancy, and currently underweight or had normal weights did not have an increased risk of type 2 diabetes. This illustrated that women who are previously diagnosed with GDM may need to be cautious about their current weight if they were overweight or obese before their pregnancies. This also emphasized that current weight status may prevail over pre-pregnancy weight status when considering the risk of type 2 diabetes.

There were several limitations in our study. First, the mean age of the study population was 52.2 years, and women were asked to report their weight when they were 18 or 20 years, which might have resulted in a recall bias. However, in a study that validated self-reported pre-pregnancy weight in a representative sample of pregnant women in the U.S. [36], the mean (standard error of the mean) difference between self-reported and imputed pre-pregnancy weight was -1.7 (0.1) kg, with an r = 0.98 (*P* <0.001) and κ = 0.78, which indicated substantial agreement. Additionally, to define type 2 diabetes, no information was available on glycated hemoglobin (HbA1c) levels and medications such as diabetic pills or insulin injection status. Lastly, a history of GDM information was based on self-reports, not proved by any blood tests.

Despite these limitations, to the best of our knowledge, the present study was the first to identify the combined effects of high pre-pregnancy BMI, high current BMI, and GDM history on the increased risk of type 2 diabetes using a large sample of Korean women (n = 59,258) in the HEXA cohort.

## Conclusions

In conclusion, elevated pre-pregnancy BMI was significantly associated with an increased risk of GDM and type 2 diabetes in Korean women. We found that women with a history of GDM who were overweight or obese before pregnancy and currently had normal weights were not associated with a higher risk of type 2 diabetes. However, women who were overweight or obese and also currently overweight or obese had a 2.54-fold increased risk of type 2 diabetes, highlighting the importance of weight management throughout the life course. Furthermore, when a woman was overweight or obese before pregnancy and remained overweight or obese about 30 years after pregnancy, there was a greater risk of developing type 2 diabetes. These findings are of great public health significance given the increasing prevalence of GDM and type 2 diabetes among Korean women. Furthermore, there is a need for the increased effort to screen for undiagnosed GDM, especially when pregnant women are overweight or obese. Interventions to achieve a healthy weight status before pregnancy and throughout the rest of one’s life may prevent the future risk of type 2 diabetes, especially in women with a history of GDM.

## Abbreviations

AOR: Adjusted odds ratio
BMI: Body mass index
CI: Confidence interval
GDM: Gestational diabetes mellitus
HEXA: Health Examinees Study
KoGES: Korean Genome and Epidemiology Study
